# Contributions of site- and sex-specific LTPs to everyday memory

**DOI:** 10.1098/rstb.2023.0223

**Published:** 2024-06-10

**Authors:** Christine M. Gall, Aliza A. Le, Gary Lynch

**Affiliations:** ^1^ Department of Anatomy and Neurobiology, University of California at Irvine, Irvine, CA 92697, USA; ^2^ Department of Neurobiology and Behavior, University of California at Irvine, Irvine, CA 92697, USA; ^3^ Department of Psychiatry and Human Behavior, University of California at Irvine, Irvine, CA 92868, USA

**Keywords:** long-term potentiation, synaptic plasticity, dentate gyrus, CA1, learning, episodic memory

## Abstract

Commentaries about long-term potentiation (LTP) generally proceed with an implicit assumption that largely the same physiological effect is sampled across different experiments. However, this is clearly not the case. We illustrate the point by comparing LTP in the CA3 projections to CA1 with the different forms of potentiation in the dentate gyrus. These studies lead to the hypothesis that specialized properties of CA1-LTP are adaptations for encoding unsupervised learning and episodic memory, whereas the dentate gyrus variants subserve learning that requires multiple trials and separation of overlapping bodies of information. Recent work has added sex as a second and somewhat surprising dimension along which LTP is also differentiated. Triggering events for CA1-LTP differ between the sexes and the adult induction threshold is significantly higher in females; these findings help explain why males have an advantage in spatial learning. Remarkably, the converse is true before puberty: Females have the lower LTP threshold and are better at spatial memory problems. A mechanism has been identified for the loss-of-function in females but not for the gain-of-function in males. We propose that the many and disparate demands of natural environments, with different processing requirements across ages and between sexes, led to the emergence of multiple LTPs.

This article is part of a discussion meeting issue ‘Long-term potentiation: 50 years on’.

## 1. Introduction

The common assertion that long-term potentiation (LTP) is the substrate for memory requires qualification. The effect can be loosely defined as a sudden and lasting increase in synaptic strength induced by a brief period of afferent activity [1,2]. However, a memory-related version of LTP would, in addition to the features included in the minimal definition, need to be triggered by conditions that actually occur during learning. Many of the stimulation protocols used in experimental work on activity-driven potentiation do not satisfy this requirement. The ‘lasting increase’ component of the definition also merits attention with regard to candidacy for LTP being a memory substrate [3,4]. Early studies of freely moving rodents showed that high-frequency stimulation of the perforant path–dentate gyrus (DG) connection produces a potentiation that lasts for weeks to months [5,6]. Similarly, theta burst stimulation (TBS) of the Schaffer-commissural (SC) afferents of CA1 was found to elicit stable potentiation that lasted for weeks ([7]; also see [4] for review). However, whether the potentiation studied in the great majority of LTP experiments is in fact long-lasting remains an open question. Relatedly, efforts to arrive at general statements about the relationship between LTP and memory need to address the likelihood that there are in fact many LTPs [8–13]. Plus, the possibility that different induction protocols trigger different cellular mechanisms and modifications within the same population of synapses has yet to be systematically tested (although coexisting variants have been described for the hippocampal mossy fibre synapse [14,15]). However, as described below, studies from a number of labs have shown that there are pronounced differences in the properties of the activity-driven synaptic plasticity at the various stages of the primary hippocampal circuit. Questions about functional significance of these plasticities, thus, need to specify the locus and the particular form of LTP that is under consideration.

The LTP-memory issue is also vague with regard to the types of memory it seeks to explain. The problem is complicated by the possibility of different processes yielding seemingly similar outcomes. Operant conditioning, which has been suggested to involve LTP [16–19], has been described in a broad range of vertebrates and invertebrates (bilaterians), which suggests that some form of the effect was present in the last common ancestor of the great majority of current animals. A recent report describes operant learning by jellyfish [20], thereby implying that a version of the effect was operational even before the bilaterians. Neurons and nervous systems are radically different across the metazoan radiation and it is not likely that the complex machinery used to produce synaptic potentiation in the mammalian hippocampus is ubiquitously distributed across this diversity. A more plausible scenario is that substrates for survival-critical operant learning evolved multiple times, somewhat in the manner proposed for eyes [21]. LTP in this case would be a specialized solution—distinguished by features such as synapse specificity and rapid induction—to a common problem. If so, then memory supported by LTP, or at least some versions of LTP, may have characteristics that distinguish it from other examples of experience-related behavioural adaptations. This local adaptation argument raises the possibility that the learning supported by LTPs is of many types, some of which may not fit into conventional psychological categories.

Here, we will evaluate evidence that a site-specific and sexually dimorphic version of potentiation plays a critical role in the encoding of information when—unlike the case for most animal studies—practice and rewards are absent. Tolman [22,23] was among the first to argue for reinforcement-free learning as an explicit alternative to the stimulus-response, behaviourist types that dominated animal psychology for much of the twentieth century. Given that the memory is formed by minimal conditions, it is reasonable to assume that its underlying mechanisms are substantially different from those used in conventional associative learning paradigms. Arguments about unsupervised learning (USL) took on greater significance when Tulving [24] advanced the persuasive argument that humans self-organize the flow of everyday experience into narrative (autobiographical) episodes. These ideas had, and continue to have, an enormous influence on research on human memory and its impairments [25]. Subsequent work showed that the hippocampus plays a central role in the acquisition and retrieval of episodes [26–30]. We will extend these analyses by showing that different elements of episodic memory are linked to specific sub-circuits within rodent hippocampus and that the LTP variant in one of these connections is critical to USL and episodic memory. As will be described, this argument also relates to the profoundly important question of whether and to what degree males and females differ in how they encode the flow of episodic experience.

## 2. Site-specific LTP in hippocampus

LTP was discovered in experiments using *in vivo* stimulation of the perforant path input to the DG middle molecular layer [31], and thus probably involved the medial perforant path. Investigations into the properties and mechanisms of LTP have since focused primarily on hippocampus, including the perforant path, but with greater emphasis on SC innervation of apical field CA1 stratum (str) radiatum in male rodents. LTP at this synapse, and its demonstrated reliance upon post-synaptic changes for both induction (*N*-methyl-d-aspartate (NMDA) receptors, calcium influx) [32,33] and expression (F-actin remodelling, increases in synapse size and AMPA receptor (AMPAR)-gated currents) [34–38], has set expectations for plasticity mechanisms at other glutamatergic synapses. Indeed, similar processes have been observed elsewhere in the cortical telencephalon [39,40]. But, as described below, recent work has shown that LTP variants quite different from that found in the CA3–CA1 connection are present at other links in the hippocampal circuit. Moreover, it now appears that the well-defined mechanisms of CA1–LTP, as elucidated in a large number of studies using male rodents, are substantially different in females. The potentiation effect is thus more differentiated, and regionally specialized, than typically thought.

### (a) Dentate gyrus

The principal afferents to the dentate gyrus (DG) granule cells terminate in largely exclusive lamina within the molecular layer; these include the lateral and medial perforant paths, that respectively innervate the outer and middle molecular layers; the commissural/associational (C/A) projections generated by the hilar mossy cells that innervate the DG inner molecular layer; and a smaller input from the supramammillary hypothalamic nucleus that terminates in a thin supragranular lamina [41]. Our recent studies of the lateral perforant path (LPP) demonstrated that the LPP–DG synapses express a form of LTP that is strikingly different from that in CA1. Potentiation in the LPP is triggered by NMDA receptors (NMDARs) and changes in post-synaptic calcium but also requires activation of metabotropic glutamate receptor 5 (mGluR5) [12] and opioid receptor-mediated suppression of GABA receptor-mediated inhibition [42,43] (among hippocampal systems the LPP and mossy fibre systems are distinctive in containing relatively high levels of opioid peptides [44,45]). Moreover, the same paired-pulse facilitation and AMPAR/NMDAR current ratio tests used to establish the post-synaptic localization of CA1-LTP [37] demonstrated that LPP–DG potentiation is expressed *pre-synaptically* by an increase in evoked transmitter release [12]. The dependency of *LPP-*LTP on mGluR5 suggested a possible explanation for how potentiation could be induced post-synaptically but expressed pre-synaptically. Specifically, the receptor is part of a supramolecular complex (signalosome) that includes diacylglycerol lipase α (DAGLα) and homer1. In association with calcium influx, the signalosome triggers synthesis of the endocannabinoid 2-arachidonoylglycerol (2-AG) [46] which is known to diffuse from the post-synaptic element to the cannabinoid type 1 receptor (CB_1_R) on axon terminals. We confirmed that 2-AG was the retrograde signal for *LPP*-DG potentiation by showing that inhibition of DAGLα or blocking or genetically ablating the CB_1_R prevented the stabilization of *LPP*-LTP [12]. Moreover, treatments that elevate 2-AG levels doubled the magnitude of *LPP-*LTP, whereas overexpressing the primary degradative enzyme blocked stabilization [12]. Using the same techniques, we found no evidence for a critical contribution of 2-AG to LTP in CA1 or in the medial perforant path (MPP)-DG system.

Besides describing a new, site-specific form of LTP, the above results were surprising because retrograde endocannabinoid signalling is known to transiently depress transmitter release at both excitatory and inhibitory synapses [47]. It follows that LPP terminals respond in a highly unusual fashion to activation of their CB_1_Rs. In line with this, studies using hippocampal slices showed that treatment with CB_1_R agonists triggers phosphorylation of vesicular fusion protein Munc18-1 at excitatory synapses in CA1, a process that would lead to Munc18-1 breakdown and the expected reduction in evoked transmitter release [48]. A similar increase in Munc18-1 phosphorylation was not evident in LPP terminals where CB_1_R agonists instead increased pre-synaptic phosphorylation of the integrin-associated focal adhesion kinase (FAK) and RhoA kinase [12]. This FAK/RhoA signalling route, which had been described for hepatocytes, provides a logical starting point for the pre-synaptic cytoskeletal changes shown in parallel experiments to underlie the enhanced release that expresses *LPP*-LTP.

It is somewhat ironic to note that the substrates for LTP in the MPP, the pathway used by Bliss and Lomo to discover LTP [31], remain poorly understood. *MPP*-LTP depends on NMDARs and post-synaptic calcium, but in contrast to the LPP, it does not rely on CB_1_R signalling [11]. In the absence of this retrograde mechanism, a post-synaptic locus seems likely. This aligns with descriptions of other post-synaptic processes influencing LTP at the MPP-DG synapse [49,50] including an interesting report suggesting that potentiation is associated with movement of NMDARs into the synaptic junction [51]. These results provide evidence for a post-synaptic locus for *MPP*-LTP, but more work is clearly needed. The same can be said for the C/A innervation of the DG inner molecular layer. These afferents from the hilar mossy cells express an NMDAR-independent, pre-synaptic form of LTP that persists for at least 1 h [13,52]. Finally, the mixed glutamatergic/GABAergic input from the supramammillary nucleus (SuM) [53,54] exhibits an exotic NMDAR-independent glutamatergic LTP that can be induced by simple post-synaptic depolarization without paired activation of the SuM afferents [55]. This passive form of potentiation of SuM input, which is induced and expressed post-synaptically, can be triggered by theta bursts delivered to the MPP. In these instances, SuM potentiation is clearly not activated in a synapse-specific manner.

In all, tests have been made for the afferents to the four zones of the DG molecular layer with a different version of potentiation found for each; none of these correspond to the well-studied form of LTP present at CA3–CA1 synapses. What is to be made of this remarkable state of affairs? Very different types of afferents are involved, with those targeting the more proximal aspect of the dendrite being somewhat unusual for the cortical telencephalon. The DG granule cells also have many peculiar features, and, from the material just discussed, this apparently extends to supporting disparate forms of synaptic potentiation. Tests for contributions of these processes to memory are lacking excepting for the perforant path. It is possible that the studies have uncovered processes that are involved in the maintenance of distinctly different types of synaptic connections but not necessarily in encoding hippocampus-dependent memories. Related to this point, and with the exception again of a small set of perforant path studies, evidence is lacking that synaptic plasticity expressed by DG afferents lasts long enough to be a substrate for anything but short-term memory.

### (b) Field CA3 and the mossy fibres

It is perhaps not surprising in light of the above that the peculiar terminals formed by granule cell projections into CA3 use an uncommon form of plasticity [14,56]. Early studies established that, unlike the case for SC projections, induction of mossy fibre (MF) potentiation caused a marked depression of paired-pulse facilitation [57] and therefore was presumably expressed by an increase in evoked transmitter release. Subsequent work showed that the induction of *MF*-LTP does not require NMDAR currents and relies on pre- but not post-synaptic calcium influx [14]. It thus bears some resemblance to potentiation of C/A input to the DG inner molecular layer. There is a second form of MF potentiation that involves the relatively small post-synaptic NMDAR currents at the MF–pyramidal cell synapse. This variant is induced and expressed post-synaptically by increased concentrations of membrane NMDARs triggered by mGluR5-mediated calcium store release [15]. There may be points of contact between these events and mechanisms of *MPP*-LTP.

The LPP and MPP projections from entorhinal cortex continue beyond the DG to densely innervate the distal-most branches of CA3 pyramidal cell dendrites [58]. Antidromic activation confirmed that the same LPP axon makes contacts on both CA3 pyramidal neurons and the outer molecular layer of the DG. However, the endocannabinoid initiated pre-synaptic potentiation found in LPP–DG contacts was altogether absent in LPP-CA3 synapses. Conventional physiological tests for enhanced release in potentiated synapses proved negative and endocannabinoid receptor antagonists had little if any effect on the induction of LTP. Conversely, intracellular application of a toxin that prevents actin polymerization disrupted the stabilization of LPP-LTP in CA3 (as it does in CA1) but not in the DG [59]. These results describe a rather startling instance in which two branches of the same input use very different forms of plasticity. Given the likelihood that the machinery needed to generate pre-synaptic LTP is transported down both branches of the LPP, we suggest that pyramidal cell spines suppress, or fail to support, events within apposed axons terminals that are needed for enhanced release. Studies have shown that MPP-CA3 synapses express NMDAR-dependent LTP [60] but little is known about substrates. This is unfortunate because comparisons between two pathways acting at two sites on the same dendrites could prove useful in extracting general rules governing the implementation of different routes to synaptic modifications.

The largest input to the CA3 pyramidal cells arises from within the subfield itself as a massive CA3 commissural–associational feedback system [61]. This system innervates well over half of the apical dendritic field and all of the extensive basal dendrites. The apical branch of the recurrent pathway exhibits NMDAR-dependent LTP in rats [62,63] and monkeys [64] but substrates have yet to be studied. Given that these are collaterals of the axons that form the CA3–CA1 connection, it is likely that many of the features of the well-defined CA1–LTP will be found in the CA3–CA3 synapses.

### (c) CA3–CA1

This is the site of the most complete effort to characterize LTP and define its substrates. Induction requires a significant degree of post-synaptic depolarization, NMDAR channel opening and increases in spine calcium [1]. The requirement for both afferent activity and post-synaptic depolarization ensures that potentiation only occurs at active synapses, so that other terminals on an axon or other spines on a dendrite are left unchanged. Movement of AMPARs into the synaptic zone—a process about which much has been learned [2,34,65]—and a concomitant increase in excitatory post synaptic currents (EPSCs) follow quickly upon the initial triggering events. Measures of paired-pulse facilitation and AMPAR/NMDAR current ratios indicate that release is unchanged [37,66,67]. Stabilization of the potentiated state involves multiple small GTPase-initiated signalling cascades, including activities triggered by brain-derived neurotrophic factor (BDNF) [68,69], resulting in reorganization of the subsynaptic actin cytoskeleton and stable expansion of the post-synaptic density [35,36,70,71]. The machinery involved overlaps with that used to form and modify adhesion junctions between various types of cells and it is thus not surprising to find that integrin signalling to the actin cytoskeleton plays a pivotal role [72–77]. There is evidence that various other types of adhesion receptors also participate in the stabilization of CA1-LTP [78,79]. Relatedly, potentiation requires calcium-driven proteolysis [1,80] and thus presumably replacement proteins. Results from studies using protein synthesis inhibitors have been controversial [81] but the bulk of the evidence indicates that local translation and induced gene expression are required for lasting potentiation [82–85].

The CA1 variant of LTP has proven particularly helpful in explaining the origins of various, seemingly unrelated features of memory. Examples include the following:

— Potentiation is induced with near-optimal efficiency by short bursts of high-frequency input spaced apart by the period of the theta rhythm (TBS), a pattern of activity often recorded during common forms of learning [86,87]. LTP is induced by only two–three naturalistic theta bursts, which relates to the very brief periods of cue sampling needed to encode memories. CA1-LTP appears to have the lowest threshold for any lasting form of potentiation thus far tested.
— TBS-induced CA1-LTP is extremely stable [4]. Potentiation was shown to endure, without decrement, for weeks in chronic recording studies that used a second set of CA3–CA1 synapses to control for the stability of the stimulation-recording arrangements [7]. The combination of low threshold for induction and extreme stability aligns well with requirements for a substrate for certain forms of memory.
— LTP has memory-like consolidation periods. A rapid, initial phase was discovered in experiments using cooling, anoxia, adenosine infusion or low-frequency stimulation after TBS [88–90]; later studies showed that rapid consolidation is dependent on actin polymerization in spines [72]. Treatments that disrupt polymerization erased LTP but only when applied within 10–15 min of induction. A second and delayed phase of consolidation was revealed with the discovery that CA1-LTP relies on transient activation and signalling by synaptic integrins and that reactivation can only be achieved after a 1 h delay. Remarkably, blocking integrins immediately prior to, but not after, their recovery eliminated previously established LTP [73,91].
— LTP expresses a ‘spaced trials’ effect [92,93]. It has been known since the nineteenth century that some forms of information are more efficiently acquired when learning sessions are conducted spaced apart rather than in a single ‘massed’ trial. Numerous explanations have been offered for the effect, among which is that some instances reflect the neurobiology of consolidation. CA1-LTP exhibits a consolidation-dependent, spaced trials effect. Specifically, a second TBS train doubles the magnitude of potentiation but only if it is delivered 1 h after the first train [92]. Two factors contribute to the effect: integrin recovery and the presence of a large population of synapses with a high plasticity threshold [91].
— The order in which afferents arrive at a CA1 dendrite determines the extent to which each will potentiate. When three small groups of fibres (A, B, C) are activated with overlapping theta bursts (B overlaps A and C overlaps B: A_1,2,3,4_, B_3,4,5,6_, C_5,6,7,8_), then A potentiates to the greatest degree and C to the least. After LTP induction, the cue A–B–C will be more likely to drive the cell than the cue C–B–A [94]. This finding could relate to the manner in which the order of the elements within a cue (e.g. phonemes within a word) is encoded by a neuron. In any event, modelling studies show that the sequence rule greatly expands the memory capacity of a CA3–CA1 type network [95].

## 3. Sex differences in LTP

### (a) Female but not male CA1-LTP is dependent on local oestrogen

**Figure 1.**
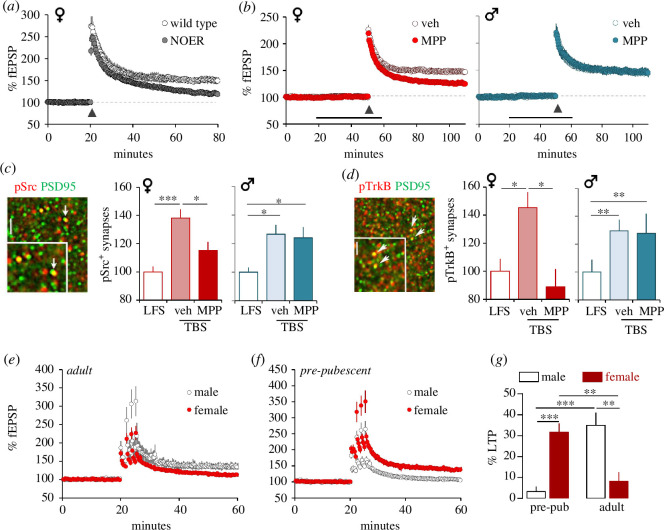
Sex differences in CA3–CA1 LTP switch over the ages of puberty. In hippocampal slices, TBS (at arrow) or low-frequency stimulation (LFS) was applied to the CA3–CA1 projections and measures were collected from CA1 str. radiatum. (*a,b*) Plots of CA3–CA1 fEPSP slopes show that stabilization of TBS-induced LTP is impaired (*a*) in female mice lacking membrane ERα (i.e. NOER mutants) and (*b*) in female, but not male, slices by infusion of ERα antagonist MPP (3 µM). (*c,d*) In slices from both males and females, TBS increases the number of post-synaptic densities (PSD95) densely double-labelled for (*c*) pSrc and (*d*) pTrkB (compared with labelling in slices receiving control LFS). Infusion of MPP attenuated (pSrc) or fully blocked (pTrkB) this increase in females only. Results are from dual-labelling immunofluorescence (shown in images) and automated fluorescence deconvolution tomography analysis (bar: 10 µm large image, 2 µm inset). (*e,f*) Plots of CA3–CA1 fEPSPs show that (*e*) in slices from adult rats TBS triplet elicits stable LTP in males but not females, whereas (*f*) the inverse was true for slices from pre-pubescent rat (pre-pub, three to four weeks old). (*g*) Plots of the percent LTP (relative to baseline, at 55–60 min post-TBS) elicited by near threshold triplet stimulation in pre-pubescent and adult rats of both sexes: this near threshold stimulation elicits LTP in females only before puberty and in males only in adulthood. Mean ± s.e.m. values plotted. *p < 0.05, **p < 0.01, ***p < 0.001. Modified from [102] for (*a*–*d*) and [104] for (*e*–*g*).

The above description of site-specific differences in LTP is based on a large collection of studies that focused almost entirely on males. There is, however, evidence for substantial sexual dimorphism in LTP. The rate-limiting enzyme (cytochrome p450 aromatase, AROM) for synthesis of oestradiol (E2), the most prevalent and potent oestrogen in brain, is abundant in hippocampus and localized to axon terminals [96–99]. E2 levels are several-fold higher in hippocampus than in blood in both sexes [98]. Both male and female hippocampal neurons release oestrogen [100]. However, as first shown by Vierk *et al*. [101] and corroborated by ourselves and others [98,102], blocking local oestrogen production with AROM inhibitors greatly reduces LTP in females only. Subsequent work using selective oestrogen receptor (ER) antagonists, and mutants that express only the membrane or nuclear forms of ERα [103], showed that membrane ERα is critical for female LTP in CA1 [102] (figure 1*a,b*). In our studies of gonadally intact rodents, none of the ERs evaluated (ERα, ERβ and GPER1) contributed to male LTP [102,105]. Relatedly, TBS-induced activation (phosphorylation) of NMDAR-linked kinases Src and ERK1/2 and of TrkB at excitatory synapses depends on ERα in females but not in males [102] (figure 1*c,d*). These results suggest that in *females only* released oestrogen ‘boosts’ kinase activation triggered by NMDAR stimulation. The ERs (α and β) directly activate the two kinases in diverse tissues [106], and increases in synaptic phosphorylated (p) Src Y418 and pERK T202/Y204 caused by 1 nM E2 infusion are dependent on ERα in females but not in males. Overall, it appears that links between ERα and LTP-critical kinases [107,108] are better developed in females than in males, thereby enabling female use of released oestrogen for synaptic modifications. Given that the NMDAR antagonist AP5 eliminates TBS-induced LTP in females as in males [34,109,110], we conclude that in females both glutamate and ERs are necessary to activate kinase signalling.

The above results indicate that some aspects of LTP-related signalling are better developed in young adult males than females so that ERα-to-kinase signalling is not required. We found no sex differences in TBS-driven depolarization and NMDAR-gated synaptic currents [104]. NMDARs are calcium permeant and their activation increases levels of the cation in spines, an effect that is required for LTP [34]. However, recent studies suggest that NMDAR-mediated Src activation involves non-ionic coupling [111–113]; a metabotropic route has also been suggested for ERK1/2 engagement [114]. Whether such effects are engaged by the minimal TBS needed to induce LTP is not known but the possibility exists that non-ionic relationships between the NMDARs and downstream kinases are better developed in males, thereby removing the need for the ERα-mediated signalling in females.

The sexually dimorphic synaptic features described above are discrete. We found no male/female differences in TBS-induced modifications of several actin management elements (e.g. β1-integrin activation, TrkB phosphorylation, cofilin phosphorylation) that stabilize LTP, although, as expected, these steps were dependent upon upstream ERα in females but not males [102].

### (b) Sex differences in adult LTP thresholds

Does the addition of a local oestrogen/ERα step in females have a significant effect on the characteristics of LTP? Initial tests of this possibility investigated the threshold amount of afferent stimulation needed to induce stable potentiation. Delivery of five pairs of theta bursts produced a robust potentiation of male CA3–CA1 synapses that showed no signs of decreasing in magnitude over a 1 h testing period [102]. In contrast, the same stimulation applied to hippocampal slices from non-pro-oestrus females failed to produce a measurable degree of potentiation. Moreover, this paired burst stimulation increased the percentage of post-synaptic densities associated with dense concentrations of pERK1/2 in the CA1 field of activated CA3 fibres in males but not in females [102]. These results suggest that the more complex machinery used by females to adjust synaptic strength is associated with an elevation in the threshold for CA1 LTP.

### (c) Sex differences in LTP reverse from before to after puberty in rodents

Sex steroid levels increase dramatically with puberty [115] raising the possibility that the oestrogen-dependent CA1-LTP in females would be weaker, or exhibit a higher threshold, before versus after puberty. We tested this using SC stimulation that was near threshold for inducing LTP in adult males: i.e. four trains of three theta bursts with 90 s between trains. Contrary to our predictions, this stimulation elicited robust LTP in pre-pubescent (four-week-old) females but not in adult females [104] (figure 1*e*,*f*). There is thus a loss-of-function during female puberty. Very different results were obtained in males: minimal TBS did not induce stable LTP in four-week-old males but was effective in young adults [104]. Thus, the threshold for LTP changes in opposite directions from before to after puberty, in the two sexes (figure 1*g*). A reasonable explanation for the female effects came with the discovery that theta burst-induced depolarization of CA1 dendrites, and NMDAR-gated responses, are much greater before than after puberty. Investigations into why the triggering events for LTP would decrease during this period uncovered a matching increase in GABAergic shunting of theta burst responses in the CA3–CA1 connection [104].

Quantitative immunofluorescence experiments did not detect puberty-related increases in the number of GABAergic synapses, as assessed by quantification of contacts immunoreactive for the scaffolding protein gephyrin, in the CA1 dendritic lamina used for the LTP experiments [104]. There was, however, a female-specific change in the GABA_A_R subunit profile over this period. Using fluorescence deconvolution tomography to quantify numbers of gephyrin+ synapses associated with GABA_A_R subunits α2, α5 and β1, we found that the number of GABAergic synapses with dense concentrations of α5 doubles from postnatal day (P) 28 to adulthood in female but not in male rat [104]. Work from other groups showed that α5-GABA_A_Rs potently shunt NMDAR-gated currents evoked by CA3 input [116]. As predicted from these results, a negative allosteric modulator for α5 containing GABA_A_Rs increased the size of theta burst responses and lowered the LTP threshold in adult females back to the low levels present immediately before the onset of puberty [104] (see below). In line with the observed weaker inhibition prior to puberty, the α5 modulator did not enhance the theta burst responses or LTP in slices from four-week-old females. These results indicate that age-related increases in inhibition, mediated by α5-containing GABA_A_Rs, are a contributor to the increase in LTP with late maturation.

The following section reviews evidence that rodents use basic elements of episodic memory and that these elements are differentially processed by various hippocampal pathways. We will then consider the argument that the distinguishing characteristics of the sexually dimorphic *CA1*-LTP are particularly appropriate for the encoding of unsupervised experience and complex episodes.

## 4. Hippocampal circuits differentially process aspects of unsupervised learning

Perhaps, the most common example of USL by rodents involves interaction with a novel environment. The animals progressively decrease exploration over a matter of minutes and search less when returned on the following day, indicating that day 1 experiences have been converted into long-term memories. The widely used object location memory (OLM) paradigm, which tests for recognizing the relocation of one of two identical objects, constitutes a second version of USL. Recently, there have been a number of efforts to test if rodents exhibit the much more complicated episodic learning, which is notable for having a temporal dimension [117,118]: events can be widely spaced apart in an episode, as when walking across a campus [119], or occur in rapid succession as in a movie [120]. This flexible use of time is especially notable given the prominent role played by temporal contiguity in conventional learning theories. Little is known about the factors occurring during or shortly after an episodic experience that promotes encoding, but emotion may have a positive effect [121]. There is also evidence that striking and unexpected input promotes storage of the *preceding* sequence of events (flashbulb memory) [122].

While not all aspects of episodic memory will be accessible to rodents, recent work suggests that learning paradigms including the following features can be used to approximate the human phenomenon:

— multiple commonplace cues or events;
— first-time encounters with a particular collection of cues;
— USL—single session with no overt rewards or particularly salient cues;
— encoding of information about the identity, location and sequence of the cues (‘what’, ‘where’ and ‘when’);
— assembling cues that are separated by either short or longer intervals into a sequence;
— novelty and emotion to promote transfer into long-term memory;
— association of an episode and its contents with the context in which they were experienced.

**Figure 2.**
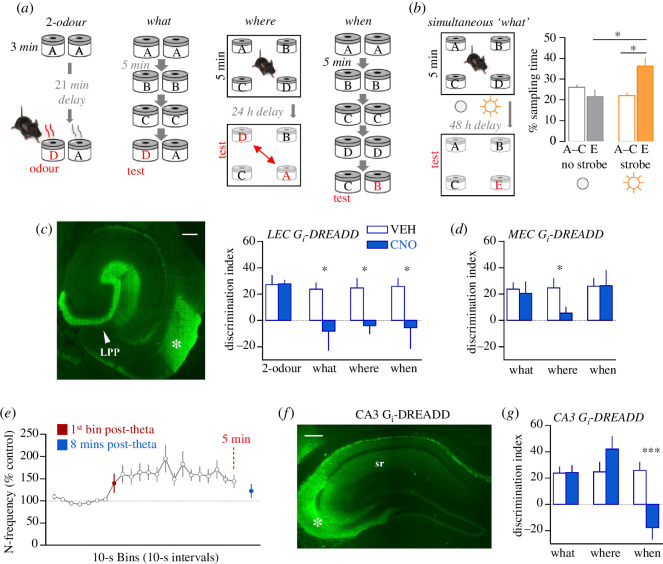
Differential involvement of hippocampal systems in acquiring the different components of episodic memory. (*a*) Illustration of paradigms used to test acquisition of cue identity (what), location (where) and temporal order (when): each task entails presentation of multiple odour cues in cups with perforated lids (letters denote specific odours) either serially (‘what’ and ‘when’) or simultaneously (where). The control ‘two-odour’ task assessed recognition of a single odour and the animals’ ability to discriminate cues. (*b*) Presentation of a mild strobe flash after initial odour exposure enhanced retention (recognition of novel cue) in a simultaneous odour episodic ‘what’ task. (*c,d,f,g*) AAV constructs mediating expression of an inhibitory Gi-DREADD (and fluorescent tag) were injected into specific subfields to enable regional silencing with clozapine N-oxide (CNO) treatment 30 min before behavioural testing approximately four weeks later. (*c*) Image shows expression of the Gi-DREADD tag in mouse LEC and its projections into the DG (arrow): bilateral LEC silencing blocked acquisition in the episodic ‘what’, ‘where’ and ‘when’ tasks. (*d*) Bilateral silencing of MEC blocked episodic ‘where’ acquisition only. (*e*) A 10-pulse theta train applied to CA3–CA1 projections elicits a prolonged period of heightened field CA1 cell firing that lasts over 5 min. (*f*) Expression of the Gi-DREADD tag in the CA3 injection site (asterisk) and projections into DG and throughout str radiatum (sr). (*g*) Unilateral silencing of a span of CA3 blocked episodic ‘when’ encoding without dampening acquisition of episodic ‘what’ or ‘where’ information. Bar in panels *c*, *f* = 400 µm. Data presented are mean ± s.e.m. *p < 0.05, ***p < 0.001. Modified from [123] and [122]. LEC, lateral entorhinal cortex; MEC, medial entorhinal cortex.

To assess this form of learning, we developed episodic learning paradigms that include most of the features on the above list [122,123]. Each paradigm entails one-time presentation of multiple cues (odours) followed by a retention trial that relies upon the animal’s native tendency to preferentially explore a novel, or least recently experienced, cue (figure 2*a*). Both mice and rats acquired information about ‘what’, ‘where’ and ‘when’ during a first-time unsupervised encounter with the cues. There was also evidence for the temporal flexibility that characterizes episodic memory. Mice recognized previously encountered odours with minutes-long intervals between cue presentations or when they sampled the odours in rapid (seconds) succession during a free-exploration period. They also remembered the *order* in which items were sampled whether the interval between cues was 30 s or 5 min [123]. It will be noted that this last result suggests that the temporal discrimination was not mediated by a simple recency effect. Both rats and mice retain information about cue identity 24 h after initial cue exposure in the free sampling (simultaneous presentation) version of the ‘what’ test but retention scores fall to chance levels by 48 h. There was, however, clear evidence for retention at 48 h when a light was flashed within 5 min of the initial cue exposure session (figure 2*b*) [122]. In other work, we found that, for male rodents, learning in the episodic paradigms is strongly dependent on context associations [124].

The hippocampal circuitry responsible for learning the components of episodic memory has not been defined. We addressed the issue by measuring retention scores in the episodic ‘what’, ‘where’ and ‘when’ paradigms and using the inhibitory DREADD approach to transiently silence specific hippocampal pathways [123]. Bilateral silencing of the lateral entorhinal cortex (LEC) during cue exposure thoroughly disrupted acquisition of ‘what’, ‘where’ and ‘when’ (figure 2*c*) without evident effect on performance in a simple two-odour memory task [123]. To test if episodic encoding specifically requires the LEC to DG connection (i.e. the LPP), we silenced LEC on one side and the DG on the other, thereby sparing non-DG LEC efferents within one hemisphere. This bilateral disconnection of the LPP fully blocked encoding in episodic ‘what’ [52]. In accord with the large body of work linking spatial information to medial entorhinal cortex (MEC), bilateral silencing of this region entirely blocked encoding of episodic ‘where’ but had no measurable influence on ‘what’ and ‘when’ (figure 2*d*) [123]. These results lead to the not surprising conclusion that data about the identity of items (LEC) are critical to all aspects of an episodic memory, whereas spatial information (MEC) is not required to learn cue identities or their temporal order.

Field CA3 was of interest with regard to ‘when’ encoding because it includes a singularly massive feedback collateral system of the type proposed by theorists to generate reverberating activity that might enable associations between items that are widely spaced in time [125]. Indeed, we have shown that a 2 s train of 5 Hz stimulation applied to the CA3 feedback collaterals produced a remarkably prolonged (minutes), self-sustained firing in approximately 40% of trials [123] (figure 2*e*). Biologically realistic simulations of CA3 suggested that such variability would occur if the pyramidal neurons underwent very large, randomly occurring depolarizations—an input arriving when a sizeable percentage of the cells happened to be partially depolarized would activate a sufficient percentage to initiate recurrent feedback within the network. Whole cell recordings confirmed that membrane potentials in CA3, but not CA1, pyramidal cells continuously undergo the dramatic (greater than or equal to 10 mV) voltage swings predicted by the modelling [123].

Experimental work then confirmed the prediction from simulations that the CA3 network with its dense interconnectivity constitutes a complex system and as such is prone to catastrophic failure. We exploited this feature to test if depression of reverberating CA3 activity affects acquisition of cue sequences. Specifically, an AAV mediating inhibitory Gi-DREADD expression was injected into a small span of CA3 pyramidal cells in one hemisphere (figure 2*f*) to depress cycling activity in the bilateral network. Administration of the DREADD agonist CNO prior to initial odour sampling did not reduce retention scores on the ‘what’ and ‘where’ tests but eliminated the discrimination between cues on the basis of their temporal order in a sequence (when) (figure 2*g*) [123].

These results reveal an unexpectedly selective association between the elements of episodic memory and sub-circuits in hippocampus: the MEC/MPP system is critical for ‘where’ encoding but not for episodic ‘what’ and ‘when’, whereas the recurrent CA3 network is needed for acquisition of episodic ‘when’ but not for encoding ‘what’ and ‘where’ information.

## 5. CA1–LTP is required for encoding of unsupervised learning

Episodic memory is encoded quickly, can persist for years (albeit in a malleable form) and often contains enormous amounts of information. As noted, LTP, as found in field CA1, expresses features that align with these points: it develops within seconds, lasts for weeks (at least) and is synapse specific. This last property, combined with empirically derived timing rules, results in tremendous storage capacity: a capability for adding new information without disturbing synaptic changes associated with earlier material. The correspondences between biological and psychological characteristics strongly suggest that the two levels of phenomena are closely related. Largely owing to the simplicity of task execution, testing the argument has often used the OLM paradigm that has features in common with those used to assess episodic learning. Animals are given a brief, one-time exposure to cues during which sampling is unsupervised (no rewards). Learning is context sensitive and dependent on hippocampal field CA1 [126]. However, the episodic features of multiple, distinctly different cues and temporal ordering are not included. OLM might thus be thought of as a partial version of an episodic memory task.

**Figure 3.**
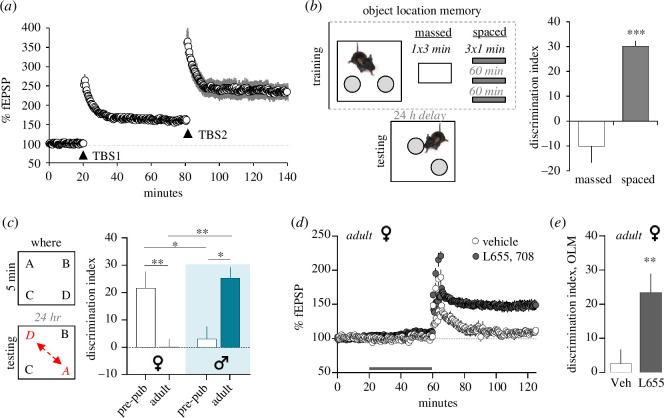
The status of CA1–LTP predicts performance in OLM and episodic memory tasks. (*a*) When spaced by 60 min, two rounds of TBS (TBS1,TBS2) each elicit comparable enhancement of the CA1 fEPSP; if spaced by 10–40 min TBS2 has no effect [91]. (*b*) In the OLM task, mice given a single 3 min object (massed) exposure fail to learn object location, whereas mice given three 1 min trials (spaced), spaced by 60 min, learn in this field CA1-dependent task [127]. (*c*) In line with age effects of CA3–CA1 LTP (in figure 1*g*), pre-pubescent female mice outperform adult females (and males) in an episodic ‘where’ task; in contrast males fail to learn in this task prior to puberty but exhibit robust learning in adulthood. (*d,e*) In line with evidence that increased inhibition dampens CA1-LTP and learning in females, treatment with a negative allosteric modulator for the α5 GABA_A_R subunit (L655,708, ‘L655’) lowers the threshold for female CA1-LTP (*d*) and learning (*e*) in the OLM task. Mean ± s.e.m. values shown; *p < 0.05, **p < 0.01, ***p < 0.001. From [104].

It was noted earlier that the unusual behaviour of synaptic integrins following their activation by TBS adds intriguing features to the stabilization of LTP that lead to non-intuitive predictions about memory consolidation. One such prediction, based on evidence for strict spacing rules for enhancement of the magnitude of LTP [92,93] with successive rounds of stimulation (figure 3*a*), is that sampling sessions spaced apart by 60 min will produce much stronger memory than sessions separated by a shorter interval. The ubiquitous spaced training effect applies to problems involving practice sessions [128] and has an uncertain relationship to the short (5–10 min) unsupervised cue exposure experiences that characterize OLM and much of episodic learning. And, there is nothing in the behavioural literature that would assign particular significance to a 1 h interval. In any event, mice given three 1 min long sampling periods separated by 1 h had excellent OLM retention at 24 h, whereas those given a single 3 min sampling period did not [127] (figure 3*b*). In further accord with the LTP timing rules, sampling sessions spaced apart by 30 min intervals did not enhance learning. Remarkably, three 20 s training sessions, again separated by 60 min, produced memory scores equivalent to those obtained with a single 5 min exposure [127]. Studies of integrin involvement in LTP uncovered a second phase of LTP stabilization that emerged between 45 and 60 min post-induction [73] (see above): infusion of β1 integrin neutralizing antisera, or agents that block protein insertion into membranes (brefeldin) during this interval—but not afterwards—caused already established LTP to decay back to baseline. Infusion of the β1 antibodies, but not IgG control solution, into field CA1 starting at 20 min after the OLM sampling phase blocked formation of long-term memory for object positions. Thus, timing rules for the substrates and magnitude of CA1–LTP led to accurate, non-intuitive predictions for OLM.

A very different type of prediction emerged from studies on the development of sex differences in LTP. As described, female rodents have a higher threshold for LTP induction than do males, and this was accompanied by a higher threshold for acquisition of episodic ‘where’ information [102,104]. However, female LTP threshold is much lower than that for males before puberty (figure 1*f*). To be consistent with the arguments for the association between LTP and encoding, the LTP threshold switch between pre-pubescent and adult animals would predict that females should outperform males on spatial problems at the younger age. There is a large literature describing male advantages on spatial problems [129–131] but the LTP results point to a specific instance in which females should have much higher retention scores. Tests of this were positive. Four-week-old female mice had excellent 24 h retention in an episodic ‘where’ paradigm, whereas age-matched males scored at chance levels; the inverse was true for adult mice (figure 3*c*) [104]. Thus, for both episodic acquisition and LTP, males undergo a marked improvement during puberty, whereas females experience a loss-of-function. Results described above indicated that the age-related adjustment to female LTP is owing to an increase in α5-GABA_A_R-mediated feedforward inhibition in CA1 (figure 3*d*). As anticipated from this, blockade of these receptors with a negative allosteric modulator for the α5 subunit restored spatial learning in adult females to levels seen before puberty [104] (figure 3*e*). In summary, LTP studies have made detailed predictions about memory that are not evident from other starting points and that have been confirmed in behavioural tests.

An alternative approach to testing for relationships between LTP and episodic-like memory is to ask if synaptic events associated with stabilization of the potentiated state are triggered by brief sessions of USL. The development of techniques for measuring TBS-induced actin signalling at individual synapses provided means for testing if learning produces similar effects. Initial studies showed that exploration of an open field causes a small but significant NMDAR-dependent increase in the percentage of synapses containing dense concentrations of *p*-cofilin in the apical dendrites of field CA1 [132]. Inactivation of the constitutively active cofilin via phosphorylation is a penultimate step towards the actin polymerization that serves to anchor synapses in their potentiated state. Synapses with high levels of *p*-cofilin were significantly larger than their neighbours [35,132]. The size of post-synaptic densities correlates with number of AMPARs and thus presumably the size of EPSCs. Subsequent work found an increase in CA1 synapses associated with activated TrkB receptor for BDNF after a period of exploration [133]; BDNF signalling is critical for the production of stable LTP by theta bursts [68,134]. It is reasonable to conclude that the signalling cascades required for LTP stabilization are set in motion by USL and produce the same structural endpoints elicited by theta bursts.

## 6. Concluding comments

The commonplace nature of USL somewhat obscures the complexity and unusual properties of the synaptic events needed for encoding. As described, rodents sampling four different odours for about half a minute while reacquainting themselves with an arena will notice if one of those odours is missing (replaced) in tests conducted the next day. More surprising still, they apparently remember the locations for each of the odours. Other experiments strongly suggest that the animals acquire information about the temporal order in which the cues were sampled. There are clear correspondences between these effects and episodic memory recorded in people [119], and it is accordingly possible that this type of learning is a characteristic feature of mammals. It is not unreasonable to assume that its acquisition was vital to the success of the group. We have argued here that there are several forms of activity-driven synaptic potentiation (LTPs) in the hippocampus and that the particular version expressed by apical CA3–CA1 synapses, and possibly many sites in cortex and amygdala, was shaped by the stringent requirements for an USL encoding device. Whether the unexpected features of CA1-LTP such as delayed consolidation [73] and the efficacy of stimulation with 1 h spacing [91,92] are also adaptations or instead consequences (exaptations) of the cell biological adjustments required to accommodate the essential features of USL is unknown. This also holds for the striking sex differences in the substrates and functional properties of CA1-LTP. What are the advantages of a higher LTP threshold in females and, relatedly, of a reversal of male–female differences in the facility for LTP during puberty? Tests are lacking but we predict that there will be aspects of USL, and episodic learning in particular, for which slower acquisition (more sampling) is an advantage. This relates to the general idea that rapid encoding can be maladaptive in noisy environments. If so, and given the further assumption of cooperative activity between males and females, then sex differences in CA1-LTP could have circumstance-dependent benefits for social groups.

While CA1-LTP aligns well with USL, it may be less than optimal for encoding the action–reward linkages that are fundamental to operant learning. There is evidence that learning new cues in a well-trained simultaneous two-cue discrimination problem activates LPP–LTP markers in the outer molecular layer of the DG [12] and that manipulating the retrograde signalling (spine to terminal) required for potentiation has the predicted consequences for operant learning [11]. A possible interpretation of these results is that the type of LTP found in the LPP is specialized so as to be sensitive to reward signals from the brainstem. Tests of whether activation of the dopaminergic inputs to the DG lowers the LPP–LTP threshold would be of interest in this regard. Another more widely discussed role for the LPP–DG system involves sharpening the distinction between inputs that have extensive overlap in their constituent elements (pattern separation) [135]. The greater number of cells in the DG than entorhinal cortex dictates that the projection from the latter to the former will be divergent, an arrangement recognized by Marr [136] as being conducive to pattern separation. Other work on sparse networks showed that LTP-based synaptic learning rules lead to categorization of cues, a process that necessarily involves the separation of inputs with shared features [137]. More generally, the long history of work investigating hippocampal contributions to behaviour suggests that the structure has multiple functional roles—if so, it would not be surprising that it uses multiple types of encoding devices. However, relating particular instances of plasticity to global operations will require analyses of how synaptic adjustments affect circuit-level operations in the hippocampus, a critically important topic about which almost nothing is known.

## Data Availability

The paper presents no new data.

## References

[1] Lynch G, Kessler M, Arai A, Larson J. 1990 The nature and causes of hippocampal long-term potentiation. Prog. Brain Res. **83**, 233–250. (10.1016/s0079-6123(08)61253-4) 2168058

[2] Nicoll RA. 2017 A brief history of long-term potentiation. Neuron **93**, 281–290. (10.1016/j.neuron.2016.12.015) 28103477

[3] Abraham WC. 2003 How long will long-term potentiation last? Philos. Trans. R. Soc. Lond. B Biol. Sci. **358**, 735–744. (10.1098/rstb.2002.1222) 12740120 PMC1693170

[4] Bliss T, Collingridge GL. 2019 Persistent memories of long-term potentiation and the N-methyl-d-aspartate receptor. Brain Neurosci. Adv. **3**, 2398212819848213. (10.1177/2398212819848213) 32166182 PMC7058229

[5] Abraham WC, Logan B, Greenwood JM, Dragunow M. 2002 Induction and experience-dependent consolidation of stable long-term potentiation lasting months in the hippocampus. J. Neurosci. **22**, 9626–9634. (10.1523/JNEUROSCI.22-21-09626.2002) 12417688 PMC6758050

[6] Barnes CA. 1979 Memory deficits associated with senescence: a neurophysiological and behavioral study in the rat. J. Comp. Physiol. Psychol. **93**, 74–104. (10.1037/h0077579) 221551

[7] Staubli U, Lynch G. 1987 Stable hippocampal long-term potentiation elicited by “theta” pattern stimulation. Brain Res. **435**, 227–234. (10.1016/0006-8993(87)91605-2) 3427453

[8] Zhou LJ *et al*. 2019 Microglia are indispensable for synaptic plasticity in the spinal dorsal horn and chronic pain. Cell Rep. **27**, 3844–3859. (10.1016/j.celrep.2019.05.087) 31242418 PMC7060767

[9] Mellor J, Nicoll RA. 2001 Hippocampal mossy fiber LTP is independent of postsynaptic calcium. Nat. Neurosci. **4**, 125–126. (10.1038/83941) 11175870

[10] Zheng F, Wess J, Alzheimer C. 2023 Long-term—but not short-term—plasticity at the mossy fiber–CA3 pyramidal cell synapse in hippocampus is altered in M1/M3 muscarinic acetylcholine receptor double knockout mice. Cells **12**, 1890. (10.3390/cells12141890) 37508553 PMC10378318

[11] Wang W *et al*. 2016 A primary cortical input to hippocampus expresses a pathway-specific and endocannabinoid-dependent form of long-term potentiation. eNeuro **3**, ENEURO.0160-16.2016. (10.1523/ENEURO.0160-16.2016) PMC4976302 27517090

[12] Wang W *et al*. 2018 Atypical endocannabinoid signaling initiates a new form of memory-related plasticity at a cortical input to hippocampus. Cereb. Cortex **28**, 2253–2266. (10.1093/cercor/bhx126) 28520937 PMC5998949

[13] Hashimotodani Y, Nasrallah K, Jensen KR, Chávez AE, Carrera D, Castillo PE. 2017 LTP at hilar mossy cell-dentate granule cell synapses modulates dentate gyrus output by increasing excitation/inhibition balance. Neuron **95**, 928–943. (10.1016/j.neuron.2017.07.028) 28817805 PMC5609819

[14] Nicoll RA, Schmitz D. 2005 Synaptic plasticity at hippocampal mossy fibre synapses. Nat. Rev. Neurosci. **6**, 863–876. (10.1038/nrn1786) 16261180

[15] Kwon HB, Castillo PE. 2008 Long-term potentiation selectively expressed by NMDA receptors at hippocampal mossy fiber synapses. Neuron **57**, 108–120. (10.1016/j.neuron.2007.11.024) 18184568 PMC2390917

[16] Gruart A, Leal-Campanario R, López-Ramos JC, Delgado-García JM. 2015 Functional basis of associative learning and its relationships with long-term potentiation evoked in the involved neural circuits: lessons from studies in behaving mammals. Neurobiol. Learn. Mem. **124**, 3–18. (10.1016/j.nlm.2015.04.006) 25916668

[17] Kishida KT, Hoeffer CA, Hu D, Pao M, Holland SM, Klann E. 2006 Synaptic plasticity deficits and mild memory impairments in mouse models of chronic granulomatous disease. Mol. Cell. Biol. **26**, 5908–5920. (10.1128/MCB.00269-06) 16847341 PMC1592752

[18] Xie X, Lu J, Ma T, Cheng Y, Woodson K, Bonifacio J, Bego K, Wang X, Wang J. 2023 Linking input- and cell-type-specific synaptic plasticity to the reinforcement of alcohol-seeking behavior. Neuropharmacology **237**, 109619. (10.1016/j.neuropharm.2023.109619) 37290535

[19] Ito W, Morozov A. 2019 Prefrontal-amygdala plasticity enabled by observational fear. Neuropsychopharmacology **44**, 1778–1787. (10.1038/s41386-019-0342-7) 30759453 PMC6785088

[20] Bielecki J, Dam Nielsen SK, Nachman G, Garm A. 2023 Associative learning in the box jellyfish Tripedalia cystophora. Curr. Biol. **33**, 4150–4159. (10.1016/j.cub.2023.08.056) 37741280

[21] Sivak JG, Sivak JM. 2019 Conserved characteristics of ocular refractive development – Did the eye evolve once? Exp. Eye Res. **183**, 84–87. (10.1016/j.exer.2018.05.007) 29758190

[22] Tolman EC. 1949 There is more than one kind of learning. Psychol. Rev. **56**, 144–155. (10.1037/h0055304) 18128182

[23] Tolman EC, Postman L. 1954 Learning. Annu. Rev. Psychol. **5**, 27–56. (10.1146/annurev.ps.05.020154.000331) 13149127

[24] Tulving E. 1984 Elements of episodic memory. Oxford, UK: Oxford University Press.

[25] Dickerson BC, Eichenbaum H. 2010 The episodic memory system: neurocircuitry and disorders. Neuropsychopharmacology **35**, 86–104. (10.1038/npp.2009.126) 19776728 PMC2882963

[26] Eichenbaum H, Sauvage M, Fortin N, Komorowski R, Lipton P. 2012 Towards a functional organization of episodic memory in the medial temporal lobe. Neurosci. Biobehav. Rev. **36**, 1597–1608. (10.1016/j.neubiorev.2011.07.006) 21810443 PMC3227798

[27] Ekstrom AD, Ranganath C. 2018 Space, time, and episodic memory: the hippocampus is all over the cognitive map. Hippocampus **28**, 680–687. (10.1002/hipo.22750) 28609014

[28] Moscovitch M, Cabeza R, Winocur G, Nadel L. 2016 Episodic memory and beyond: the hippocampus and neocortex in transformation. Annu. Rev. Psychol. **67**, 105–134. (10.1146/annurev-psych-113011-143733) 26726963 PMC5060006

[29] Smith DM, Mizumori SJY. 2006 Hippocampal place cells, context, and episodic memory. Hippocampus **16**, 716–729. (10.1002/hipo.20208) 16897724

[30] Rolls ET. 2022 The hippocampus, ventromedial prefrontal cortex, and episodic and semantic memory. Prog. Neurobiol. **217**, 102334. (10.1016/j.pneurobio.2022.102334) 35870682

[31] Bliss TVP, Lomo T. 1973 Long-lasting potentiation of synaptic transmission in the dentate area of the anesthetized rabbit following stimulation of the perforant path. J Physiol. **232**, 334–356. (10.1113/jphysiol.1973.sp010273) PMC1350458 4727084

[32] Coan EJ, Saywood W, Collingridge GL. 1987 MK-801 blocks NMDA receptor-mediated synaptic transmission and long term potentiation in rat hippocampal slices. Neurosci. Lett. **80**, 111–114. (10.1016/0304-3940(87)90505-2) 2821457

[33] Kumar A. 2011 Long-term potentiation at CA3-CA1 hippocampal synapses with special emphasis on aging, disease, and stress. Front. Aging Neurosci. **3**, 7. (10.3389/fnagi.2011.00007) 21647396 PMC3102214

[34] Granger AJ, Nicoll RA. 2014 Expression mechanisms underlying long-term potentiation: a postsynaptic view, 10 years on. Philos. Trans. R. Soc. Lond. B Biol. Sci. **369**, 20130136. (10.1098/rstb.2013.0136) 24298139 PMC3843869

[35] Chen LY, Rex CS, Casale MS, Gall CM, Lynch G. 2007 Changes in synaptic morphology accompany actin signaling during LTP. J. Neurosci. **27**, 5363–5372. (10.1523/JNEUROSCI.0164-07.2007) 17507558 PMC6672340

[36] Lynch G, Rex CS, Gall CM. 2007 LTP consolidation: substrates, explanatory power, and functional significance. Neuropharmacology **52**, 12–23. (10.1016/j.neuropharm.2006.07.027) 16949110

[37] Muller D, Lynch G. 1988 Long-term potentiation differentially affects two components of synaptic responses in hippocampus. Proc. Natl Acad. Sci. USA **85**, 9346–9350. (10.1073/pnas.85.23.9346) 2904150 PMC282736

[38] Lisman J, Yasuda R, Raghavachari S. 2012 Mechanisms of CaMKII action in long-term potentiation. Nat. Rev. Neurosci. **13**, 169–182. (10.1038/nrn3192) 22334212 PMC4050655

[39] Maren S. 1999 Long-term potentiation in the amygdala: a mechanism for emotional learning and memory. Trends Neurosci. **22**, 561–567. (10.1016/s0166-2236(99)01465-4) 10542437

[40] Gavin CF, Rubio MD, Young E, Miller C, Rumbaugh G. 2012 Myosin II motor activity in the lateral amygdala is required for fear memory consolidation. Learn. Mem. **19**, 9–14. (10.1101/lm.024042.111) 22174310 PMC3246591

[41] Amaral DG, Witter MP. 1995 Hippocampal formation. In The rat nervous system (ed. G Paxinos), pp. 443–486, 2 edn. San Diego, CA: Academic Press.

[42] Bramham CR, Sarvey JM. 1996 Endogenous activation of μ and δ-1 opioid receptors is required for long-term potentiation induction in the lateral perforant path: dependence on GABAergic inhibition. J. Neurosci. **16**, 8123–8131. (10.1523/JNEUROSCI.16-24-08123.1996) 8987837 PMC6579214

[43] Breindl A, Derrick BE, Rodriguez SB, Martinez JL. 1994 Opioid receptor-dependent long-term potentiation at the lateral perforant path-CA3 synapse in rat hippocampus. Brain Res. Bull. **33**, 17–24. (10.1016/0361-9230(94)90045-0) 8275323

[44] Gall C, Brecha N, Karten HJ, Chang KJ. 1981 Localization of enkephalin-like immunoreactivity to identified axonal and neuronal populations of the rat hippocampus. J. Comp. Neurol. **198**, 335–350. (10.1002/cne.901980211) 6263955

[45] Gall C. 1984 Ontogeny of dynorphin-like immunoreactivity in the hippocampal formation of the rat. Brain Res. **307**, 327–331. (10.1016/0006-8993(84)90487-6) 6147175

[46] Piomelli D. 2014 More surprises lying ahead. The endocannabinoids keep us guessing. Neuropharmacology **76**, 228–234. (10.1016/j.neuropharm.2013.07.026) 23954677 PMC3855347

[47] Castillo PE, Younts TJ, Chávez AE, Hashimotodani Y. 2012 Endocannabinoid signaling and synaptic function. Neuron **76**, 70–81. (10.1016/j.neuron.2012.09.020) 23040807 PMC3517813

[48] Schmitz SK *et al*. 2016 Presynaptic inhibition upon CB1 or mGlu2/3 receptor activation requires ERK/MAPK phosphorylation of Munc18-1. EMBO J. **35**, 1236–1250. (10.15252/embj.201592244) 27056679 PMC4888233

[49] Leal G *et al*. 2017 The RNA-binding protein hnRNP K mediates the effect of BDNF on dendritic mRNA metabolism and regulates synaptic NMDA receptors in hippocampal neurons. eNeuro **4**, ENEURO.0268-17.2017. (10.1523/ENEURO.0268-17.2017) PMC5732018 29255796

[50] Cooke SF *et al*. 2006 Autophosphorylation of αCaMKII is not a general requirement for NMDA receptor-dependent LTP in the adult mouse. J. Physiol. **574**, 805–818. (10.1113/jphysiol.2006.111559) 16728448 PMC1817742

[51] Harney SC, Jane DE, Anwyl R. 2008 Extrasynaptic NR2D-containing NMDARs are recruited to the synapse during LTP of NMDAR-EPSCs. J. Neurosci. **28**, 11685–11694. (10.1523/JNEUROSCI.3035-08.2008) 18987204 PMC3844786

[52] Wang W *et al*. 2018 Treating a novel plasticity defect rescues episodic memory in fragile X model mice. Mol. Psychiatry **23**, 1798–1806. (10.1038/mp.2017.221) 29133950 PMC5951717

[53] Vertes RP. 2015 Major diencephalic inputs to the hippocampus: supramammillary nucleus and nucleus reuniens. Circuitry and function. Prog. Brain Res. **219**, 121–144. (10.1016/bs.pbr.2015.03.008) 26072237 PMC4961211

[54] Gall C, Selawski L. 1984 Supramammillary afferents to guinea pig hippocampus contain substance P-like immunoreactivity. Neurosci. Lett. **51**, 171–176. (10.1016/0304-3940(84)90546-9) 6083512

[55] Tabuchi E, Sakaba T, Hashimotodani Y. 2022 Excitatory selective LTP of supramammillary glutamatergic/GABAergic cotransmission potentiates dentate granule cell firing. Proc. Natl Acad. Sci. USA **119**, e2119636119. (10.1073/pnas.2119636119) 35333647 PMC9060512

[56] Makani S, Lutzu S, Lituma PJ, Hunt DL, Castillo PE. 2021 Retrograde suppression of post-tetanic potentiation at the mossy fiber-CA3 pyramidal cell synapse. eNeuro **8**, ENEURO.0450-20.2021. (10.1523/ENEURO.0450-20.2021) PMC7986537 33593734

[57] Staubli U, Larson J, Lynch G. 1990 Mossy fiber potentiation and long-term potentiation involve different expression mechanisms. Synapse **5**, 333–335. (10.1002/syn.890050410) 2360200

[58] Witter MP. 1993 Organization of the entorhinal-hippocampal system: a review of current anatomical data. Hippocampus **3**, 33–44. (10.1002/hipo.1993.4500030707) 8287110

[59] Quintanilla J, Jia Y, Pruess BS, Chavez J, Gall CM, Lynch G, Gunn BG. 2024 Pre- versus post-synaptic forms of LTP in two branches of the same hippocampal afferent. J. Neurosci. **44**, e1449232024. (10.1523/JNEUROSCI.1449-23.2024) 38326038 PMC10919254

[60] Do VH, Martinez CO, Martinez JL, Derrick BE. 2002 Long-term potentiation in direct perforant path projections to the hippocampal CA3 region in vivo. J. Neurophysiol. **87**, 669–678. (10.1152/jn.00938.2000) 11826036

[61] Witter MP. 2007 Intrinsic and extrinsic wiring of CA3: Indications for connectional heterogeneity. Learn. Mem. **14**, 705–713. (10.1101/lm.725207) 18007015

[62] Martinez CO, Do VH, Martinez JL, Derrick BE. 2002 Associative long-term potentiation (LTP) among extrinsic afferents of the hippocampal CA3 region in vivo. Brain Res. **940**, 86–94. (10.1016/s0006-8993(02)02598-2) 12020879

[63] Zalutsky RA, Nicoll RA. 1990 Comparison of two forms of long-term potentiation in single hippocampal neurons. Science **248**, 1619–1624. (10.1126/science.2114039) 2114039

[64] Urban NN, Henze DA, Lewis DA, Barrionuevo G. 1996 Properties of LTP induction in the CA3 region of the primate hippocampus. Learn. Mem. **3**, 86–95. (10.1101/lm.3.2-3.86) 10456079

[65] Díaz-Alonso J, Nicoll RA. 2021 AMPA receptor trafficking and LTP: carboxy-termini, amino-termini and TARPs. Neuropharmacology **197**, 108710. (10.1016/j.neuropharm.2021.108710) 34271016 PMC9122021

[66] Muller D, Lynch G. 1989 Evidence that changes in presynaptic calcium currents are not responsible for long-term potentiation in hippocampus. Brain Res. **479**, 290–299. (10.1016/0006-8993(89)91631-4) 2924160

[67] Kauer JA, Malenka RC, Nicoll RA. 1988 A persistent postsynaptic modification mediates long-term potentiation in the hippocampus. Neuron **1**, 911–917. (10.1016/0896-6273(88)90148-1) 2908443

[68] Rex CS, Lin CY, Kramár EA, Chen LY, Gall CM, Lynch G. 2007 Brain-derived neurotrophic factor promotes long-term potentiation-related cytoskeletal changes in adult hippocampus. J. Neurosci. **27**, 3017–3029. (10.1523/JNEUROSCI.4037-06.2007) 17360925 PMC6672589

[69] Korte M, Kang H, Bonhoeffer T, Schuman E. 1998 A role for BDNF in the late-phase of hippocampal long-term potentiation. Neuropharmacology **37**, 553–559. (10.1016/s0028-3908(98)00035-5) 9704996

[70] Rex CS, Chen LY, Sharma A, Liu J, Babayan AH, Gall CM, Lynch G. 2009 Different Rho GTPase-dependent signaling pathways initiate sequential steps in the consolidation of long-term potentiation. J. Cell Biol. **186**, 85–97. (10.1083/jcb.200901084) 19596849 PMC2712993

[71] Rex CS *et al*. 2010 Myosin IIb regulates actin dynamics during synaptic plasticity and memory formation. Neuron **67**, 603–617. (10.1016/j.neuron.2010.07.016) 20797537 PMC2929390

[72] Kramár EA, Lin B, Rex CS, Gall CM, Lynch G. 2006 Integrin-driven actin polymerization consolidates long-term potentiation. Proc. Natl Acad. Sci. USA **103**, 5579–5584. (10.1073/pnas.0601354103) 16567651 PMC1459396

[73] Babayan AH *et al*. 2012 Integrin dynamics produce a delayed stage of long-term potentiation and memory consolidation. J. Neurosci. **32**, 12 854–12 861. (10.1523/JNEUROSCI.2024-12.2012) 22973009 PMC3752079

[74] Chan C-S, Weeber EJ, Kurup S, Sweatt JD, Davis RL. 2003 Integrin requirement for hippocampal synaptic plasticity and spatial memory. J. Neurosci. **23**, 7107–7116. (10.1523/JNEUROSCI.23-18-07107.2003) 12904471 PMC6740650

[75] Chun D, Gall CM, Bi X, Lynch G. 2001 Evidence that integrins contribute to multiple stages in the consolidation of long term potentiation in rat hippocampus. Neuroscience **105**, 815–829. (10.1016/s0306-4522(01)00173-7) 11530220

[76] Staubli U, Vanderklish PW, Lynch G. 1990 An inhibitor of integrin receptors blocks LTP. Behav. Neural Biol. **53**, 1–5. (10.1016/0163-1047(90)90712-f) 2154174

[77] Wang X, Bozdagi O, Nikitczuk JS, Zhai ZW, Zhou Q, Huntley GW. 2008 Extracellular proteolysis by matrix metalloproteinase-9 drives dendritic spine enlargement and long-term potentiation coordinately. Proc. Natl Acad. Sci. USA **105**, 19 520–19 525. (10.1073/pnas.0807248105) PMC2614793 19047646

[78] Castillo PE. 2022 Unique transsynaptic complexes enable long-term synaptic plasticity in a synapse-specific manner. Proc. Natl Acad. Sci. USA **119**, e2206429119. (10.1073/pnas.2206429119) 35737826 PMC9271206

[79] Bozdagi O, Shan W, Tanaka H, Benson DL, Huntley GW. 2000 Increasing numbers of synaptic puncta during late-phase LTP: N-cadherin is synthesized, recruited to synaptic sites, and required for potentiation. Neuron **28**, 245–259. (10.1016/s0896-6273(00)00100-8) 11086998

[80] Vanderklish P, Bednarski E, Lynch G. 1996 Translational suppression of calpain blocks long-term potentiation. Learn. Mem. **3**, 209–217. (10.1101/lm.3.2-3.209) 10456091

[81] Lynch G, Kramár EA, Gall CM. 2015 Protein synthesis and consolidation of memory-related synaptic changes. Brain Res. **1621**, 62–72. (10.1016/j.brainres.2014.11.060) 25485773

[82] Fonseca R, Nägerl UV, Bonhoeffer T. 2006 Neuronal activity determines the protein synthesis dependence of long-term potentiation. Nat. Neurosci. **9**, 478–480. (10.1038/nn1667) 16531998

[83] Guzowski JF, Lyford GL, Stevenson GD, Houston FP, McGaugh JL, Worley PF, Barnes CA. 2000 Inhibition of activity-dependent arc protein expression in the rat hippocampus impairs the maintenance of long-term potentiation and the consolidation of long-term memory. J. Neurosci. **20**, 3993–4001. (10.1523/JNEUROSCI.20-11-03993.2000) 10818134 PMC6772617

[84] Sajikumar S, Navakkode S, Frey JU. 2007 Identification of compartment- and process-specific molecules required for “synaptic tagging” during long-term potentiation and long-term depression in hippocampal CA1. J. Neurosci. **27**, 5068–5080. (10.1523/JNEUROSCI.4940-06.2007) 17494693 PMC6672381

[85] Stanton PK, Sarvey JM. 1984 Blockade of long-term potentiation in rat hippocampal CA1 region by inhibitors of protein synthesis. J. Neurosci. **4**, 3080–3088. (10.1523/JNEUROSCI.04-12-03080.1984) 6502226 PMC6564864

[86] Larson J, Wong D, Lynch G. 1986 Patterned stimulation at the theta frequency is optimal for the induction of hippocampal long-term potentiation. Brain Res. **368**, 347–350. (10.1016/0006-8993(86)90579-2) 3697730

[87] Otto T, Eichenbaum H, Wiener SI, Wible CG. 1991 Learning-related patterns of CA1 spike trains parallel stimulation parameters optimal for inducing hippocampal long-term potentiation. Hippocampus **1**, 181–192. (10.1002/hipo.450010206) 1669292

[88] Larson J, Xiao P, Lynch G. 1993 Reversal of LTP by theta frequency stimulation. Brain Res. **600**, 97–102. (10.1016/0006-8993(93)90406-d) 8422592

[89] Arai A, Larson J, Lynch G. 1990 Anoxia reveals a vulnerable period in the development of long-term potentiation. Brain Res. **511**, 353–357. (10.1016/0006-8993(90)90184-d) 2334854

[90] Arai A, Kessler M, Lynch G. 1990 The effects of adenosine on the development of long-term potentiation. Neurosci. Lett. **119**, 41–44. (10.1016/0304-3940(90)90750-4) 2097583

[91] Lynch G, Kramár EA, Babayan AH, Rumbaugh G, Gall CM. 2013 Differences between synaptic plasticity thresholds result in new timing rules for maximizing long-term potentiation. Neuropharmacology **64**, 27–36. (10.1016/j.neuropharm.2012.07.006) 22820276 PMC3445784

[92] Kramár EA, Babayan AH, Gavin CF, Cox CD, Jafari M, Gall CM, Rumbaugh G, Lynch G. 2012 Synaptic evidence for the efficacy of spaced learning. Proc. Natl Acad. Sci. USA **109**, 5121–5126. (10.1073/pnas.1120700109) 22411798 PMC3323981

[93] Cao G, Harris KM. 2014 Augmenting saturated LTP by broadly spaced episodes of theta-burst stimulation in hippocampal area CA1 of adult rats and mice. J. Neurophysiol. **112**, 1916–1924. (10.1152/jn.00297.2014) 25057146 PMC4200006

[94] Larson J, Lynch G. 1989 Theta pattern stimulation and the induction of LTP: the sequence in which synapses are stimulated determines the degree to which they potentiate. Brain Res. **489**, 49–58. (10.1016/0006-8993(89)90007-3) 2743153

[95] Granger R, Whitson J, Larson J, Lynch G. 1994 Non-Hebbian properties of long-term potentiation enable high-capacity encoding of temporal sequences. Proc. Natl Acad. Sci. USA **91**, 10104–10108. (10.1073/pnas.91.21.10104) 7937845 PMC44966

[96] Naftolin F, Horvath TL, Jakab RL, Leranth C, Harada N, Balthazart J. 1996 Aromatase immunoreactivity in axon terminals of the vertebrate brain. An immunocytochemical study on quail, rat, monkey and human tissues. Neuroendocrinology **63**, 149–155. (10.1159/000126951) 9053779

[97] Peterson RS, Yarram L, Schlinger BA, Saldanha CJ. 2005 Aromatase is pre-synaptic and sexually dimorphic in the adult zebra finch brain. Proc. Biol. Sci. **272**, 2089–2096. (10.1098/rspb.2005.3181) 16191621 PMC1559905

[98] Hojo Y, Kawato S. 2018 Neurosteroids in adult hippocampus of male and female rodents: biosynthesis and actions of sex steroids. Front. Endocrinol. **9**, 183. (10.3389/fendo.2018.00183) PMC5925962 29740398

[99] Kato A, Hojo Y, Higo S, Komatsuzaki Y, Murakami G, Yoshino H, Uebayashi M, Kawato S. 2013 Female hippocampal estrogens have a significant correlation with cyclic fluctuation of hippocampal spines. Front. Neural Circuits **7**, 149. (10.3389/fncir.2013.00149) 24151456 PMC3798982

[100] Fester L, Prange-Kiel J, Zhou L, Blittersdorf BV, Böhm J, Jarry H, Schumacher M, Rune GM. 2012 Estrogen-regulated synaptogenesis in the hippocampus: sexual dimorphism in vivo but not in vitro. J. Steroid Biochem. Mol. Biol. **131**, 24–29. (10.1016/j.jsbmb.2011.11.010) 22138012

[101] Vierk R *et al*. 2012 Aromatase inhibition abolishes LTP generation in female but not in male mice. J. Neurosci. **32**, 8116–8126. (10.1523/JNEUROSCI.5319-11.2012) 22699893 PMC6703647

[102] Wang W, Le AA, Hou B, Lauterborn JC, Cox CD, Levin ER, Lynch G, Gall CM. 2018 Memory-related synaptic plasticity Is sexually dimorphic in rodent hippocampus. J. Neurosci. **38**, 7935–7951. (10.1523/JNEUROSCI.0801-18.2018) 30209204 PMC6136152

[103] Pedram A, Razandi M, Lewis M, Hammes S, Levin ER. 2014 Membrane-localized estrogen receptor alpha is required for normal organ development and function. Dev. Cell **29**, 482–490. (10.1016/j.devcel.2014.04.016) 24871949 PMC4062189

[104] Le AA, Lauterborn JC, Jia Y, Wang W, Cox CD, Gall CM, Lynch G. 2022 Prepubescent female rodents have enhanced hippocampal LTP and learning relative to males, reversing in adulthood as inhibition increases. Nat. Neurosci. **25**, 180–190. (10.1038/s41593-021-01001-5) 35087246 PMC8876130

[105] Wang W, Kantorovich S, Babayan AH, Hou B, Gall CM, Lynch G. 2016 Estrogen’s effects on excitatory synaptic transmission entail integrin and TrkB transactivation and depend upon β1-integrin function. Neuropsychopharmacology **41**, 2723–2732. (10.1038/npp.2016.83) 27272766 PMC5026741

[106] Fu XD, Simoncini T. 2008 Extra-nuclear signaling of estrogen receptors. IUBMB Life **60**, 502–510. (10.1002/iub.80) 18618586

[107] Salter MW. 1998 Src, N-methyl-D-aspartate (NMDA) receptors, and synaptic plasticity. Biochem. Pharmacol. **56**, 789–798. (10.1016/s0006-2952(98)00124-5) 9774140

[108] Kelleher RJ, Govindarajan A, Jung HY, Kang H, Tonegawa S. 2004 Translational control by MAPK signaling in long-term synaptic plasticity and memory. Cell **116**, 467–479. (10.1016/s0092-8674(04)00115-1) 15016380

[109] Collingridge GL, Singer W. 1990 Excitatory amino acid receptors and synaptic plasticity. Trends Pharmacol. Sci. **11**, 290–296. (10.1016/0165-6147(90)90011-v) 2167544

[110] Bliss TVP, Collingridge GL, Morris RGM. 2003 Introduction. Long-term potentiation and structure of the issue. Philos. Trans. R. Soc. Lond. B Biol. Sci. **358**, 607–611. (10.1098/rstb.2003.1282) 12740102 PMC1693168

[111] Weilinger NL *et al*. 2016 Metabotropic NMDA receptor signaling couples Src family kinases to pannexin-1 during excitotoxicity. Nat. Neurosci. **19**, 432–442. (10.1038/nn.4236) 26854804

[112] Gray JA, Zito K, Hell JW. 2016 Non-ionotropic signaling by the NMDA receptor: controversy and opportunity. F1000Res. **5**, F1000 Faculty Rev-1010. (10.12688/f1000research.8366.1) PMC4882754 27303637

[113] Le AA, Lauterborn JC, Jia Y, Cox CD, Lynch G, Gall CM. 2024 Metabotropic NMDA receptor signaling contributes to sex differences in synaptic plasticity and episodic memory. bioRxiv 2024.01.26.577478. (10.1101/2024.01.26.577478) PMC11638816 39424366

[114] Yang L, Mao L, Tang Q, Samdani S, Liu Z, Wang JQ. 2004 A novel Ca^2+^-independent signaling pathway to extracellular signal-regulated protein kinase by coactivation of NMDA receptors and metabotropic glutamate receptor 5 in neurons. J. Neurosci. **24**, 10 846–10 857. (10.1523/JNEUROSCI.2496-04.2004) PMC6730215 15574735

[115] Vetter-O’Hagen CS, Spear LP. 2012 Hormonal and physical markers of puberty and their relationship to adolescent-typical novelty-directed behavior. Dev. Psychobiol. **54**, 523–535. (10.1002/dev.20610) 21953609 PMC3288810

[116] Schulz JM, Knoflach F, Hernandez MC, Bischofberger J. 2018 Dendrite-targeting interneurons control synaptic NMDA-receptor activation via nonlinear α5-GABA_A_ receptors. Nat. Commun. **9**, 3576. (10.1038/s41467-018-06004-8) 30177704 PMC6120902

[117] Chao OY, de Souza Silva MA, Yang YM, Huston JP. 2020 The medial prefrontal cortex - hippocampus circuit that integrates information of object, place and time to construct episodic memory in rodents: behavioral, anatomical and neurochemical properties. Neurosci. Biobehav. Rev. **113**, 373–407. (10.1016/j.neubiorev.2020.04.007) 32298711 PMC7302494

[118] Eacott MJ, Easton A. 2010 Episodic memory in animals: remembering which occasion. Neuropsychologia **48**, 2273–2280. (10.1016/j.neuropsychologia.2009.11.002) 19913043

[119] Dede AJO, Frascino JC, Wixted JT, Squire LR. 2016 Learning and remembering real-world events after medial temporal lobe damage. Proc. Natl Acad. Sci. USA **113**, 13480–13485. (10.1073/pnas.1617025113) 27821761 PMC5127365

[120] Tang H *et al*. 2016 Predicting episodic memory formation for movie events. Sci. Rep. **6**, 30175. (10.1038/srep30175) 27686330 PMC5043190

[121] Zlomuzica A, Preusser F, Totzeck C, Dere E, Margraf J. 2016 The impact of different emotional states on the memory for what, where and when features of specific events. Behav. Brain Res. **298**, 181–187. (10.1016/j.bbr.2015.09.037) 26548361

[122] Quintanilla J, Cox BM, Gall CM, Mahler SV, Lynch G. 2021 Retrograde enhancement of episodic learning by a postlearning stimulus. Learn. Mem. **28**, 82–86. (10.1101/lm.052191.120) 33593926 PMC7888236

[123] Cox BM, Cox CD, Gunn BG, Le AA, Inshishian VC, Gall CM, Lynch G. 2019 Acquisition of temporal order requires an intact CA3 commissural/associational (C/A) feedback system in mice. Commun. Biol. **2**, 251. (10.1038/s42003-019-0494-3) 31286068 PMC6610080

[124] Le AA, Palmer LC, Chavez J, Gall CM, Lynch G. 2024 Sex differences in the context dependency of episodic memory. Front. Behav. Neurosci. **18**, 1349053. (10.3389/fnbeh.2024.1349053) 38516050 PMC10956361

[125] Hebb DO. 1949 The organization of behavior. New York, NY: Wiley.

[126] Barrett RM, Malvaez M, Kramár EA, Matheos DP, Arrizon A, Cabrera SM, Lynch G, Greene RW, Wood MA. 2011 Hippocampal focal knockout of CBP affects specific histone modifications, long-term potentiation, and long-term memory. Neuropsychopharmacology **36**, 1545–1556. (10.1038/npp.2011.61) 21508930 PMC3138668

[127] Seese RR, Wang K, Yao YQ, Lynch G, Gall CM. 2014 Spaced training rescues memory and ERK1/2 signaling in fragile X syndrome model mice. Proc. Natl Acad. Sci. USA **111**, 16907–16912. (10.1073/pnas.1413335111) 25385607 PMC4250145

[128] Smolen P, Zhang Y, Byrne JH. 2016 The right time to learn: mechanisms and optimization of spaced learning. Nat. Rev. Neurosci. **17**, 77–88. (10.1038/nrn.2015.18) 26806627 PMC5126970

[129] Jones CM, Braithwaite VA, Healy SD. 2003 The evolution of sex differences in spatial ability. Behav. Neurosci. **117**, 403–411. (10.1037/0735-7044.117.3.403) 12802870

[130] Barel E, Tzischinsky O. 2018 Age and sex differences in verbal and visuospatial abilities. Adv. Cogn. Psychol. **2**, 51–61. (10.5709/acp-0238-x) 32362962 PMC7186802

[131] Andreano JM, Cahill L. 2009 Sex influences on the neurobiology of learning and memory. Learn. Mem. **16**, 248–266. (10.1101/lm.918309) 19318467

[132] Fedulov V, Rex CS, Simmons DA, Palmer L, Gall CM, Lynch G. 2007 Evidence that long-term potentiation occurs within individual hippocampal synapses during learning. J. Neurosci. **27**, 8031–8039. (10.1523/JNEUROSCI.2003-07.2007) 17652593 PMC6672739

[133] Chen LY, Rex CS, Pham DT, Lynch G, Gall CM. 2010 BDNF signaling during learning is regionally differentiated within hippocampus. J. Neurosci. **30**, 15097–15101. (10.1523/JNEUROSCI.3549-10.2010) 21068315 PMC3073526

[134] Edelmann E, Cepeda-Prado E, Franck M, Lichtenecker P, Brigadski T, Lessmann V. 2015 Theta burst firing recruits BDNF release and signaling in postsynaptic CA1 neurons in spike-timing-dependent LTP. Neuron **86**, 1041–1054. (10.1016/j.neuron.2015.04.007) 25959732

[135] Reagh ZM, Yassa MA. 2014 Object and spatial mnemonic interference differentially engage lateral and medial entorhinal cortex in humans. Proc. Natl Acad. Sci. USA **111**, E4264–E4273. (10.1073/pnas.1411250111) 25246569 PMC4210036

[136] Marr D. 1971 Simple memory: a theory for archicortex. Phil. Trans. R. Soc. Lond. B **262**, 23–81. (10.1098/rstb.1971.0078) 4399412

[137] Ambros-Ingerson J, Granger R, Lynch G. 1990 Simulation of paleocortex performs hierarchical clustering. Science **247**, 1344–1348. (10.1126/science.2315702) 2315702

